# Screening the components of Saussurea involucrata for novel targets for the treatment of NSCLC using network pharmacology

**DOI:** 10.1186/s12906-021-03501-0

**Published:** 2022-02-28

**Authors:** Dongdong Zhang, Tieying Zhang, Yao Zhang, Zhongqing Li, He Li, Yueyang Zhang, Chenggong Liu, Zichao Han, Jin Li, Jianbo Zhu

**Affiliations:** School of Life Sciences, Shihezi University, Xiangyang street, Shihezi, 832003 PR China

**Keywords:** Saussurea involucrata, LUAD (Lung adenocarcinoma), LUSC (Lung squamous cell carcinoma), Non-small cell lung cancer, Molecular dynamics

## Abstract

**Background:**

Saussurea involucrata (SAIN), also known as Snow lotus (SI), is mainly distributed in high-altitude areas such as Tibet and Xinjiang in China. To identify novel targets for the prevention or treatment of lung adenocarcinoma and lung squamous cell carcinoma (LUAD&LUSC), and to facilitate better alternative new drug discovery as well as clinical application services, the therapeutic effects of SAIN on LUAD&LUSC were evaluated by gene differential analysis of clinical samples, compound target molecular docking, and GROMACS molecular dynamics simulation.

**Results:**

Through data screening, alignment, analysis, and validation it was confirmed that three of the major active ingredients in SAIN, namely quercetin (Q), luteolin (L), and kaempferol (K), mainly act on six protein targets, which mainly regulate signaling pathways in cancer, transcriptional misregulation in cancer, EGFR tyrosine kinase inhibitor resistance, adherens junction, IL-17 signaling pathway, melanoma, and non-small cell lung cancer. In addition, microRNAs in cancer exert preventive or therapeutic effects on LUAD&LUSC. Molecular dynamics (MD) simulations of Q, L, or K in complex with EGFR, MET, MMP1, or MMP3 revealed the presence of Q in a very stable tertiary structure in the human body.

**Conclusion:**

There are three active compounds of Q, L, and K in SAIN, which play a role in the treatment and prevention of non-small cell lung cancer (NSCLC) by directly or indirectly regulating the expression of genes such as MMP1, MMP3, and EGFR.

## Introduction

In 2021, among many types of cancers, lung cancer is still among the tough, not only because its mortality is as high as 22% in both men and women [1], but also because its treatment is challenging.

According to the microscopic morphological characteristics of lung cancer cells, lung cancer can be divided into two types: small cell lung cancer (SCLC) and non-small cell lung cancer (NSCLC) [2]. In previous studies, it has been shown that the two-year survival rate of SCLC is very low, hovering around 14% [2]. Fortunately, the two-year survival rate of NSCLC can reach 42% [2]. In addition, NSCLC accounts for 85% of all cases of lung cancer, which is good news for investigators in cancer-targeted therapy and NSCLC patients [3].

Currently, the world's targeted therapeutics for NSCLC have issues, including high R&D cost, a relatively expensive market price, and a long administration cycle. Therefore, these restrictions do not allow every NSCLC patient to use these drugs normally. Lung adenocarcinoma (LUAD) and lung squamous cell carcinoma (LUSC) are the two most common types of NSCLC, among which, LUAD accounts for far more cases than LUSC, and has become the major type of targeted therapy for NSCLC.

Saussurea involucrata (SAIN) is mainly distributed in high altitude areas such as Xinjiang and Tibet in China. Research on the pharmacological effects of SAIN started in 1980 [4] and has continued for more than 40 years now. There are many known pharmacological activities of SAIN, but the most studied effects are its anti-inflammatory, antioxidant [5], and anticancer (prostate, gastric, and breast cancer, etc.) effects [6]. The effects of SAIN on other diseases (e.g., lung cancer) have not yet been studied. Therefore, studies are urgently needed to evaluate and supplement the efficacy of SAIN for the treatment of lung cancer.

Network pharmacology is a new discipline that is based on the theory of systems biology to select specific signal nodes for multi-target drug molecule design [7]. Network pharmacology emphasizes the multi pathway regulation of signaling pathways to improve the therapeutic efficacy of drugs and to reduce toxic side effects, thereby improving the success rate of clinical trials of novel drugs and saving the cost of drug research and development [7]. Network pharmacology is a newly emerging research method in recent years, which was first proposed by Professor Hopkins, a pharmacologist at Dundee University, United Kingdom [7]. Therefore, network pharmacology methods were used, combining systems pharmacology, gene differential analysis, molecular docking, subcellular localization prediction, patient prognosis survival analysis, and molecular dynamics simulation. In this study, the mechanism of action of the targets for the treatment and prevention of LUAD and LUSC by the active ingredients in SAIN.

## Materials and Methods

### Screening of active ingredients with drug resistance in SAIN

Traditional Chinese Medicine Systems Pharmacology Database (TCMSP) (https://tcmsp-e.com/) [8] is one of the most commonly used databases for active ingredient screening of traditional Chinese medicine. The advantage of TCMSP lies in that the parameters of oral bioavailability (OB) and drug likeness (DL) are available from this database. OB and DL are important for the evaluation of drug efficacy, only when the OB exceeds a certain value (OB ≥ 30%) and the DL is within a certain range (DL ≥ 0.18) when only able to effectively reflect the class resistance of a certain ingredient.

Among them, the DL value for this system is calculated following Eq. (1), and to obtain the drug of interest, the DL value is functional only if the lead compound is chemically easily synthesized and has the properties of absorption, distribution, metabolism, excretion (ADME).

1 $$T(x,y)=\frac{\mathrm{x}-\mathrm{y}}{{\mid x\mid }^{2}+{\mid y\mid }^{2}-xy}$$

In the equation, x represents the descriptive index of all ingredients in SAIN, and y represents the descriptive index from the drug bank (https://www.drugbank.ca)database [9] for the average drug similarity index of an ingredient.

The lipid water partition coefficient $\mathrm{log}{P}_{O/W}$ refers to the partition coefficient of the drug in the n-octanol–water system and is widely used as a measure of the hydrophobicity of chemical constituents. This represents the main driving force for effective ingredient permeation through biological membranes composed of lipid bilayers, thereby controlling the ingredient target binding effect. The calculation of $\mathrm{log}{P}_{O/W}$ is presented in Eq. (2):

2 $$log{P}_\frac{O}{W}=\frac{\mathrm{log}{C}_{O}}{\mathrm{log}{C}_{W}}$$

In the equation, C_O_ represents the equilibrium concentration of the drug in the oil phase and C_W_ represents the equilibrium concentration of the drug in the aqueous phase. The magnitude of the $\mathrm{log}{P}_{O/W}$ value represents the magnitude of solute hydrophobicity. The larger the $\mathrm{log}{P}_{O/W}$, the stronger the hydrophobicity and vice versa. Therefore, $\mathrm{log}{P}_{O/W}\le 5$ was used as the screening criterion.

Target prediction and construction of an active ingredient target network.

The structural formula of the active ingredients of SAIN obtained above (Fig. 5c) was plotted on the STP (http://www.swisstargetprediction.ch) database [10] for target prediction. To obtain the ultimately required disease target information, the predicted results were merged with the NSCLC targets retrieved on the TCMSP database. Next, Cytoscape 3.8.0 software [11] was used for visual construction of the ingredient target network (C – T network) for the above active ingredients and targets to obtain the C – T network relationship diagram. Herein, a primary screen for lung cancer-related active ingredients of SAIN was completed.

### Gene differential analysis and gene TPM data analysis for LUAD&LUSC

To further screen the active ingredients and their targets that were initially screened, a large number of clinical case samples of LUAD&LUSC were obtained from the GEO (https://www.ncbi.nlm.nih.gov/geo/) database [12]. Differential gene analyses of ten thousand samples, which were randomly selected samples used to draw the differential heat map of relevant genes, were performed for the primary screening of targets. To make the secondary screening of targets more explicit, gene IDs of targets were used to draw a quantitative scatter plot of transcripts per million (TPM) of the screened genes based on the TCGA data provided by the GEPIA (http://gepia.cancer-pku.cn/) database [13]. In addition, the effective ingredients and their action targets for the treatment of LUAD&LUSC in SAIN were further screened out.

### Gene enrichment analysis, construction of protein–protein interaction network, and ingredients-targets pathway network

To evaluate the mutual influence among the targets screened above, a protein interaction network and an active ingredient target pathway network were constructed. The biological processes and pathways that each target participates in in vivo were explored, and data of target genes were retrieved through the David (https://david.ncifcrf.gov/) database [14]. Biological process (BP) analysis and Kyoto Encyclopedia of Genes and Genomes (KEGG) [15] enrichment analysis in Gene Ontology (GO) analysis were performed. Of these, FDR ≤ 0.05 for GO analysis and FDR ≤ 0.05 for KEGG enrichment analysis, both meet the requirement of genes to be significantly enriched in vivo were statistically significant.

The FDR value is a corrected p-value, and the results screened out with FDR are also more precise. Therefore, this step aims to obtain the biological process and in vivo pathway of target action, which will provide the basis for subsequent studies.

### Molecular docking and subcellular localization prediction

Molecular docking was performed by tools, including Autodock 4.2.6 software [16] and PyMol software [17]. In brief, the binding energy size of ingredients to targets was first used to verify whether the effect of the active ingredients from SAIN in treating LUAD&LUSC was reliable, and to exclude the ingredients from SAIN that were less effective on LUAD&LUSC.

Molecular docking is an approach for drug design that uses the characteristics of receptors and the mode of interaction between receptors and drug molecules [18]. One theoretical simulation approach is to primarily study the interactions between molecules, such as ligands and receptors, and to predict their binding modes and avidities. In recent years, the molecular docking method has become an important technique in the field of computer-assisted drug research [19].

In recent years, subcellular localization prediction has been a more popular method of subcellular localization. Existing data to create a database of the sequence relationships of the target sequences and subcellular structures of various genes and their regulation can accurately predict the location of the target protein on various organelles and cell membranes, and has multiple advantages. Currently used subcellular localization prediction tools include (1) the PSORT II (https://psort.hgc.jp/form2.html) database [20], which uses the k-nearest neighbor (k-NN) algorithm, a commonly used learning algorithm in data mining and machine learning. K-NN is widely used, and PSORT II can be used to identify a classical nuclear localization signal (CNLS) sequence. Accuracy of PSORT II is very high under conditions with a large sample size [21]; (2) The CELLO (http://cello.life.nctu.edu.tw/) database [22] uses the SVM-RFE algorithm(Support vector machine recursive feature elimination) to construct ranking coefficient based on weight vector w generated by SVM at training. IT removes one feature attribute with the smallest ranking coefficient for each iteration, thereby finally ranking all feature attributes in decreasing order [23]; (3) The BUSCA (http://busca.biocomp.unibo.it/) database [24] employs the betaware algorithm and aims to solve the detection of TMBB (transmembrane beta-barrels) in proteomes and the prediction of its topology [24, 25].

Combined with the analysis of biological processes, using PSORT II, CELLO, and BUSCA databases for subcellular location prediction, respectively, and to compare the prediction results of the three tools, the intersection part was selected and the main location of the target protein in the cell was obtained.

### Prognostic survival analysis of patients

In clinical studies, to evaluate the efficacy of a drug and know the survival data, such as the survival time of patients after surgery, our analysis of these survival data is called survival analysis.

After a series of screening and analysis of the ingredients and action targets of SAIN, prognostic overall survival analysis was performed on the gene IDs of the targets of the final active ingredient actions in SAIN, so as to explore the length of time that patients can survive when drugs act on these targets over time.

### Molecular dynamics simulation validation by GROMACS

Based on molecular docking studies, differential gene analysis, and the prediction of subcellular localization and survival analysis, the results were validated with molecular dynamics (MD) simulations, a means to computationally model the motion of a small molecule in an organismal environment [26].

MD simulations were performed with GROMACS software [27], thereby setting the physical conditions to a constant temperature (305 K), constant pressure (101 kPa), and periodic boundary conditions to simulate the human body using the TIP3P water model in a neutral sodium chloride solution of 0.145 M.

After state equilibration of all environments, we performed 50 ns MD simulations of the ingredient target complex system using epidermal growth factor receptor (EGFR) with L, Q, matrix metalloproteinase 3 (MMP3) with Q, hepatocyte growth factor receptor (MET) with L, and matrix metalloproteinase 1 (MMP1) with L, Q, and K, in which stored conformations were calculated every 10 ps (1 s = 10^12^ ps). In addition, the root mean square deviation (RMSD) of the MD simulation results analysis and visualization were performed using a GROMACS inbuilt program with the RMSD formula as follows (3).

3 $$RMSD=\sqrt{\frac1N{\textstyle\sum_{i=1}^{i=N}}{(R_t-R_{ref})}^2}$$

In this equation, R_t_-R_Ref_ represents the position of the t-th atom at a certain frame minus its position in the reference conformation ref (positional offset), and N refers to the number of atoms.

## Results

### Screening results of active ingredients in SAIN

In this study, a total of 55 known active ingredients in SAIN were collected from the TCMSP database, including alkaloids, lipids, flavonoids, and flavonoids. Subsequently, six potent drug-resistant ingredients from 55 compounds were screened under the following screening conditions of OB ≥ 30%&DL ≥ 0.18&logPo/w ≤ 5. Details of the screening process of these six components, Flazin (F), Q, K, L, Alloisoimperatorin (A), and Hispidulin (H), are shown in Table 1.

**Table 1:** Screening information of six compounds in SAIN

Molecule Name	MW(KDa)	logP(o/w)	OB(%)	DL	PubChem CID
Flazin	308.31	3.235	94.27575085	0.38559	5,377,686
Quercetin	302.25	1.504	46.43334812	0.27525	5,280,343
Kaempferol	286.25	1.771	41.88224954	0.24066	5,280,863
Luteolin	286.25	2.067	36.16262934	0.24552	5,280,445
Alloisoimperatorin	270.3	3.793	34.80406732	0.21854	5,317,436
Hispidulin	300.28	2.318	30.97205344	0.27025	5,281,628

### Results of targets screening and ingredients-targets network analysis

**Fig. 1:**
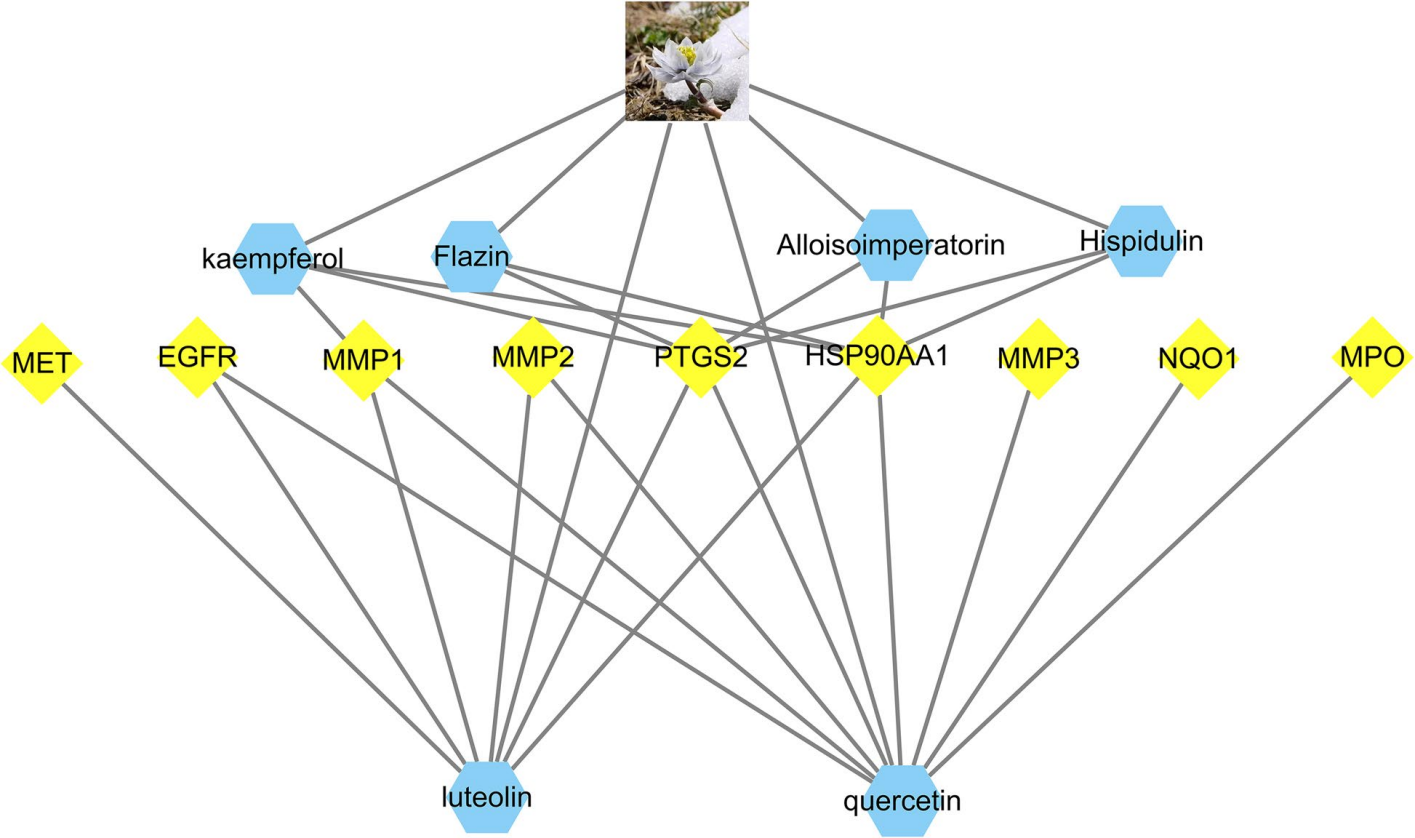
C-T network, the benzene ring represents the compounds and the diamond represents the targets

By merging 1023 targets screened from the STP database and 764 LUAD and LUSC targets screened from the TCMSP database, a total of 9 targets with six ingredient roles was obtained. The nine protein targets interacted with the ingredients of six SAIN, and an ingredient target network was constructed using Cytoscape, resulting in an ingredient target network characterized by 15 nodes and 24 edges (Fig. 1).

As determined by the C-T network, the ranking of six components with strong and weak target effects of SAIN was as follows: Q, L, K, and H, F, and A. Moreover, the rank order of the nine target and component effects was as follows: prostaglandin endoperoxide synthase 2 (PTGS2), 90 kDa heat shock protein ΑA1 (Hsp90AA1), MMP1, and EGFR.

### Differential analysis of genes and TPM data analysis results

To ensure the accuracy of the experimental data, further screening for ingredients and targets was performed. In this study, data from a total of 22,589 LUAD and 22,697 LUSC related disease genes was obtained through the GEO database and R 4.0.4, TCGA data through the GDC (https://portal.gdc.cancer.gov/) database [28]. A total of 535 LUAD samples and 59 LUAD control group samples was obtained, respectively, and 502 LUSC samples and 49 LUSC control samples were obtained. Undeniably, these data are all highly informative[28].

**Fig. 2:**
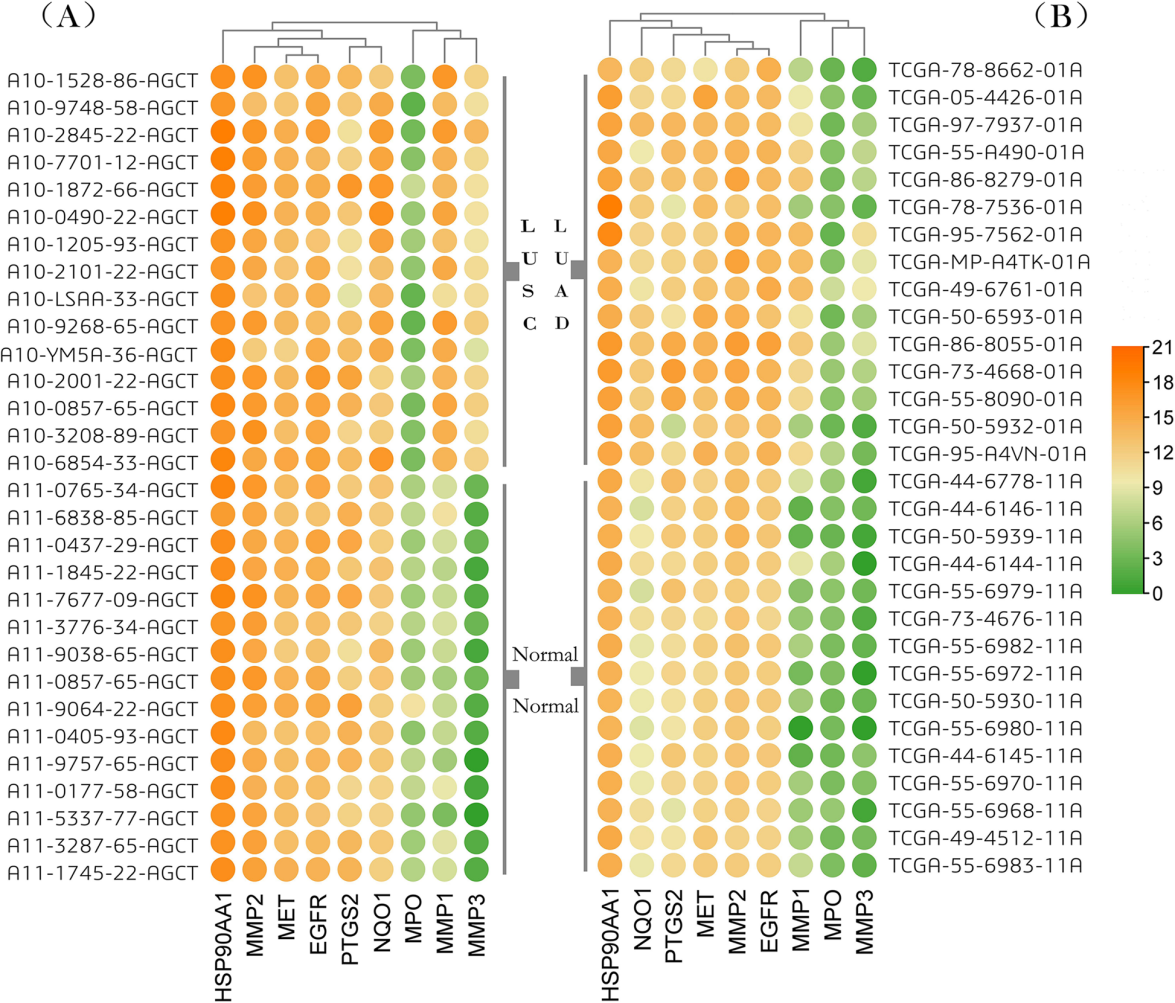
Heat map: gene difference analysis of LUAD & LUSC, Green indicates lower expression, orange is the opposite. **a** 30 clinical samples of LUSC corresponding to 9 targets (including 15 normal controls) **b** 30 clinical samples of LUAD corresponding to 9 targets (including 15 normal controls)

To make the experiment go smoothly, data were selected by combining the targets and clinical samples using R language with the nine targets under study as the targets of interest by using the p-value range (P ≤ 0.05) of these data and the patient samples to obtain the gene expression matrix of two groups, LUAD & Normal and LUSC & Normal, corresponding to the nine targets. In this study, 15 samples from each of the two groups within the two cohorts were selected, including 30 clinical samples from each of the LUSC & control group in LUAD and LUSC, and the gene differential heat maps of LUAD (Fig. 2B) and LUSC (Fig. 2a The direction of the ordinate is “ ← ”) were plotted using TBtools v1.082 software [29]. Since the nine targets can act on both types of lung cancer, a gene cluster was created, since our samples were divided into two groups (experimental and normal), to make the sample dispersion uniform, and without affecting the experimental results, we do not cluster the samples.

**Fig. 3:**
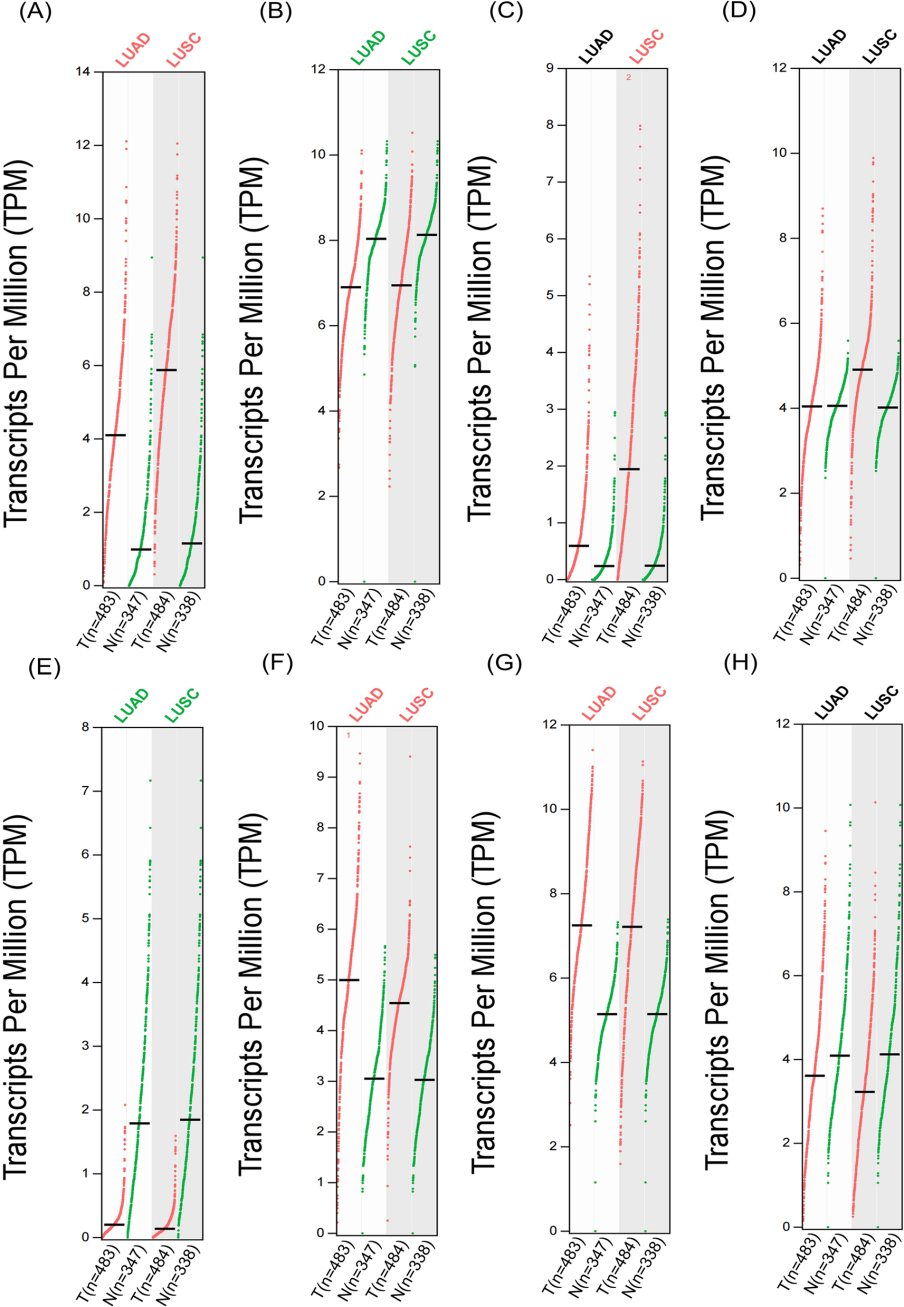
Gene TPM analysis of 8 targets of LUAD and LUSC. **a **MMP1. **b **MMP2.  **c **MMP3.  **d **EGFR.  **e **MPO. **f **MET. **g **NQO1.  **h **PTGS2

The results of gene difference analysis showed that the difference of Hsp90AA1 between LUAD and LUSC was least obvious, therefore, this was discarded. The gene IDs of the remaining eight targets were TPM analyzed by GEPIA database, input gene ID, one-way analysis of variance (ANOVA) with Log_2_FC value, and Q value of 1 and 0.01 were selected by the difference method [30], and the total number of involved samples were as follows: LUAD (T = 483, N = 347), LUSC (T = 486, N = 338), in which the ‘T’ represented tumor patients and the ‘N’ represented normal controls (Fig. 3).

When combining the correspondence between components and targets, and the results of gene difference analysis and TPM analysis, the data showed that the expression and changes of MMP2 and PTGS2 in group T were consistent with that in group N, therefore, they were discarded.

The SAIN components with major effects on LUAD and LUSC were Q, l, and K, and the genes with major effects on LUAD and LUSC were MMP1, MMP3, EGFR, MET, NAD (P) H: quinone oxidoreductase 1 (NQO1), and myeloperoxidase (MPO).

For the analysis of six genes, we concluded that the expression of two genes, EGFR and MMP3, were much more effective in LUSC than in LUAD, whereas MET had the opposite effect. Although the other three genes (MMP1, NQO1 and MPO) showed consistent expression in the occurrence and development of LUAD and LUSC, MPO was of interest as this gene showed full-stage high expression in cases in the N group and low expression in cases in the T group. Therefore, we speculated that overexpression of MPO in tissues might be effective in inhibiting the development of LUAD and LUSC.

### GO (BP), KEGG enrichment analysis, PPI analysis, and C-T pathway analysis results

Based on the above-mentioned analysis, three targets, included Hsp90AA1, PTGS2, and MMP2 were excluded, and previous studies have shown that Hsp90AA1 and the isotype Asp90AB1 may be required for the treatment of two subtypes of breast cancer (MCF-7 and BT-474) [31]. PTGS2 is required in diseases, such as colorectal cancer [32], while MMP2 mostly plays a role in inflammatory responses and immune responses [33].

**Fig. 4:**
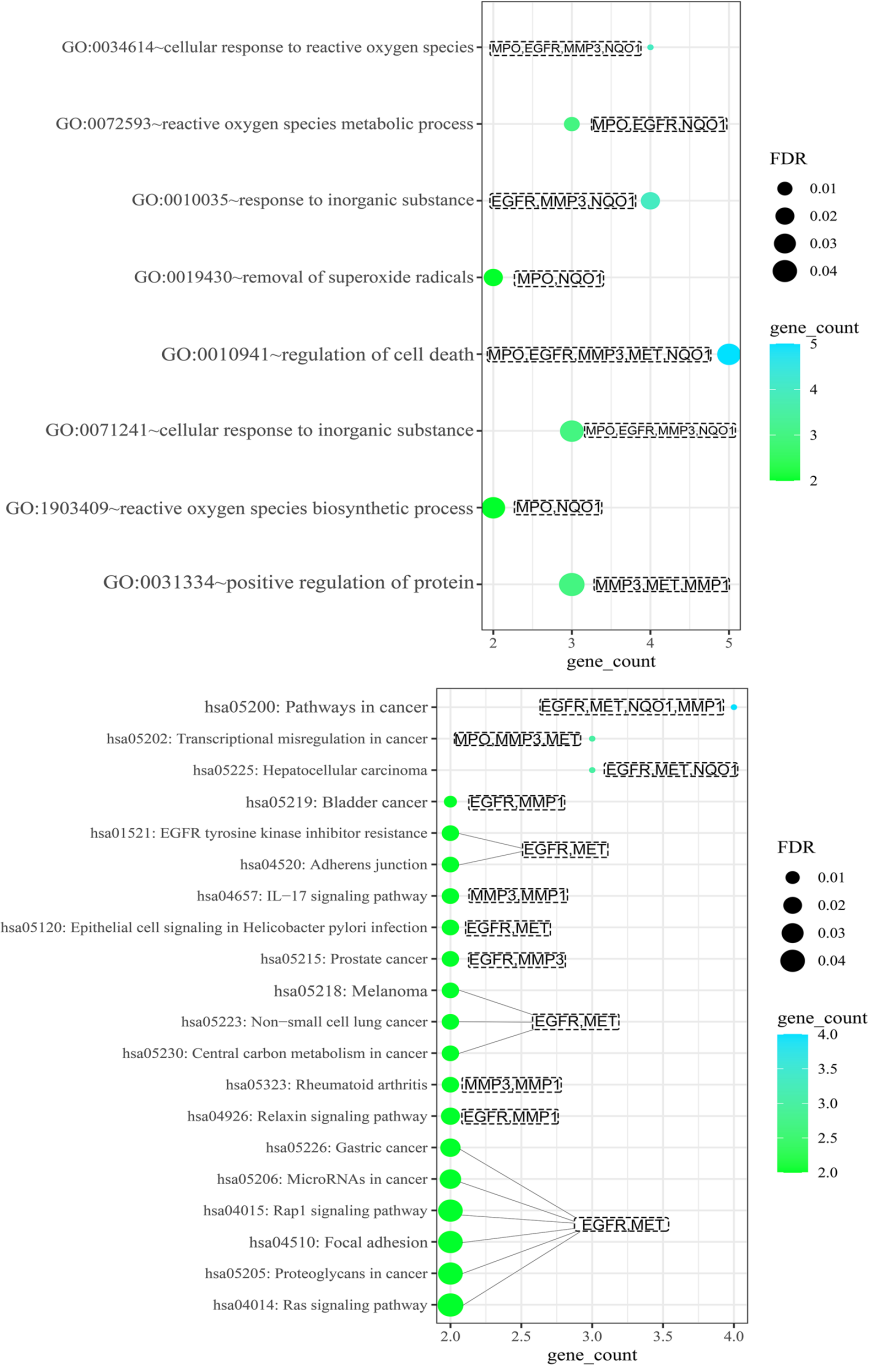
BP analysis and KEGG analysis of 8 targets affected by SAIN, the size of the target is represented by the counts of participating targets (**a**). BP analysis of secondary screened targets (**b**). KEGG analysis of secondary screened targets

**Fig. 5:**
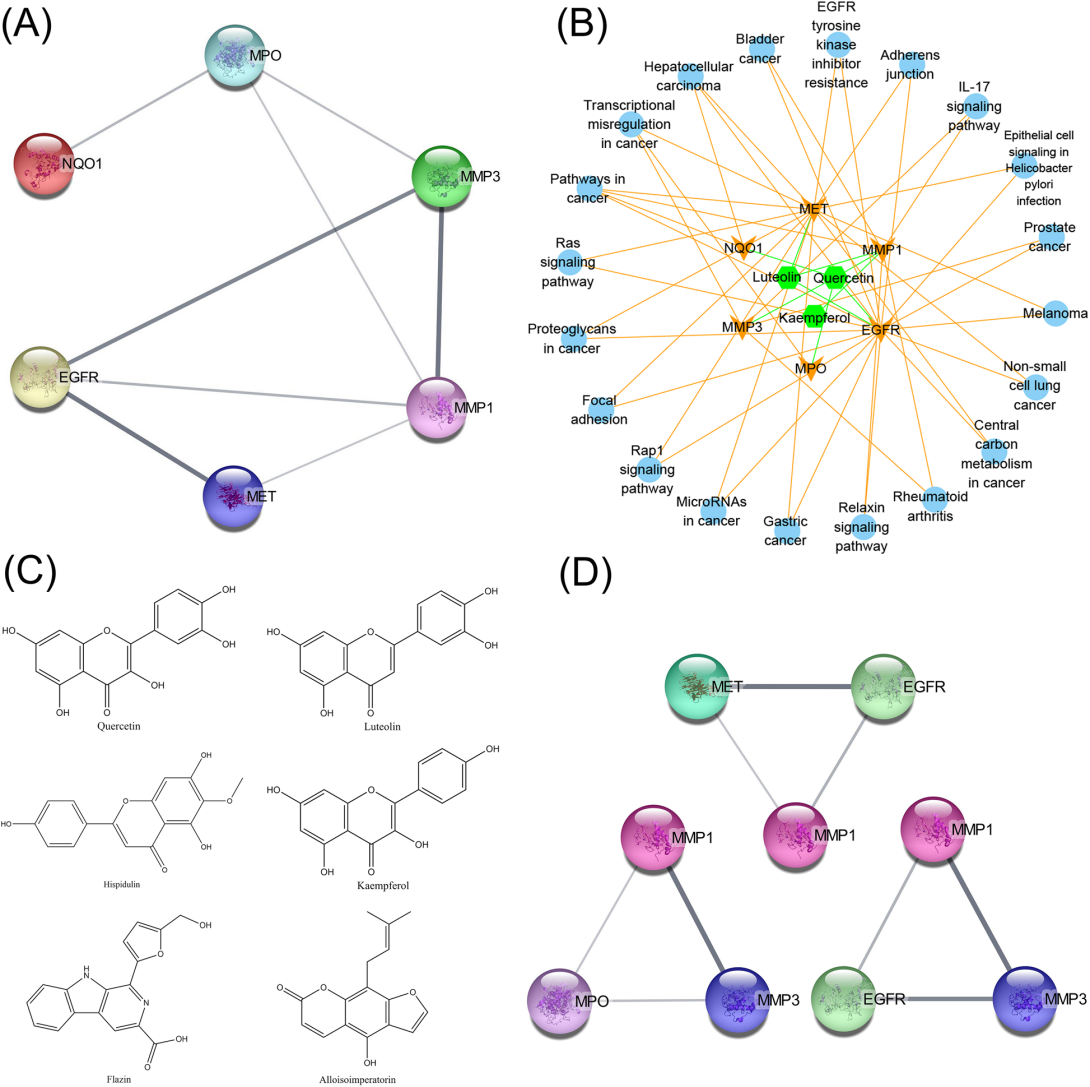
**a** Eight protein interaction networks mediated by six components of SAIN (**b**). Components-targets-pathways network (**c**). Structure of six components in SAIN (**d**). Interaction of MMP1 with EGFR, MMP3, MET and MPO

The remaining six targets (EGFR, MET, MMP1, MMP3, NQO1 and MPO) were subjected to undergo BP analysis (Fig. 4a), KEGG enrichment analysis (Fig. 4b) [15], and PPI (protein protein interaction) analysis (Fig. 5a).

To achieve credibility and statistical significance of the data, we performed GO (BP) analysis and KEGG enrichment analysis at FDR ≤ 0.05. Referring to the results of KEGG enrichment analysis, and to make it easy to understand, we used Cytoscape to associate the three components, six targets, and 20 pathways to draw a component target pathway diagram (Fig. 5b). This diagram shows the relationship between components and targets. The relationship between targets and pathways is visualized, and a total of 20 metabolic regulatory pathways, including 29 nodes and 53 edges were obtained.

First, from the C-T-P (compounds-target-pathway) network diagram, targets were sorted into EGFR, MET, MMP1, MMP3, NQO1, and MPO based on the number of regulatory pathways and corresponding components, and pathways were ranked into pathways in cancer, transcriptional misregulation in cancer, hepatocellular carcinoma, bladder cancer, and EGFR tyrosine kinase inhibitor resistance. According to the degree of correlation adherens junction, IL-17 signaling pathway, melanoma, non-small cell lung cancer, relaxin signaling pathway and microRNAs in cancer, etc. However, although these pathways contribute to both LUAD and LUSC, the strength of the contribution is still indistinguishable, and therefore, further analysis and validation (immunofluorescence, etc.) are needed.

### Analysis of molecular docking results

After a series of analysis, screening, and validation efforts as mentioned above, we identified the target sites where the three components (L, Q and K) act separately, where the three components can act on multiple targets, where, Q can act on MMP1, MMP3, EGFR, MPO, and NQO1; L can act on MMP1, EGFR, and MET; and K only acts on MMP1.

**Fig. 6:**
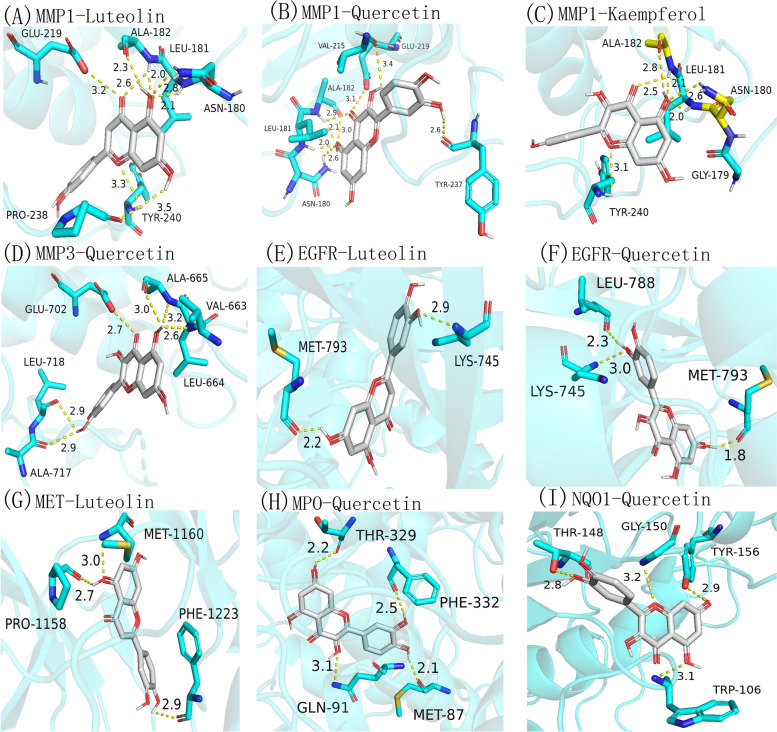
Molecular docking results of 6 components and 8 proteins, the connection represents a hydrogen bond. **a **L-MMP1. **b **Q-MMP1. **c **K-MMP1. **d **Q-MMP3. e L-EGFR. **f **Q-EGFR. **g** L-MET. **h** Q-MPO. **i **Q-NQO1

After obtaining this information, the structures of the 6 components were plotted using ChemDraw ultra 12.0 software [34], saved as mol2 files, and the corresponding tertiary structures of proteins with ligands were selected according to the structures of the components using the PDB (https://www.rcsb.org/) database [35]. PDB files were downloaded, and the PDB protein files were processed by discovery studio 4.5 client software [36] (to remove water molecules and excess structures, etc.). Both file formats were saved as pdbqt files using autodock 1.5.6 software, ligands and coordinate positions (x, y, z) were selected for component screening and ranked by the distance of hydrogen bonds. Tertiary structures were predicted using an X-ray diffusion method, and the resolution of 6 proteins was the lowest at 1.90 Å and the highest at 2.40 Å. Next, Autodock 1.5.6 and Autodock Vina were used for molecular docking of ingredients with targets, and finally, the docking results were processed using PyMol (Fig. 6).

The coordinates of each component in the tertiary structure are EGFR (12,12,14), MET (16,14,12), MMP1 (12,12,12), MMP3 (16,16,16), MPO (16,14,10), and NQO1 (22,22,22). The larger the value in this coordinate, the greater the affinity for docking, and the greater the affinity (interactions existing between ligand and receptor), thereby demonstrating that the tighter the binding of the component to the target, the more effective the component is. However, to guarantee the availability and reference value of the data, we set the coordinates (x, y, z) according to the structure size of the ingredients. Therefore, the coordinate values that were used in this study were appropriate for the ingredients. The best value was − 10.1 kcal/mol and the worst was − 7.9 kcal/mol.

**Fig. 7:**
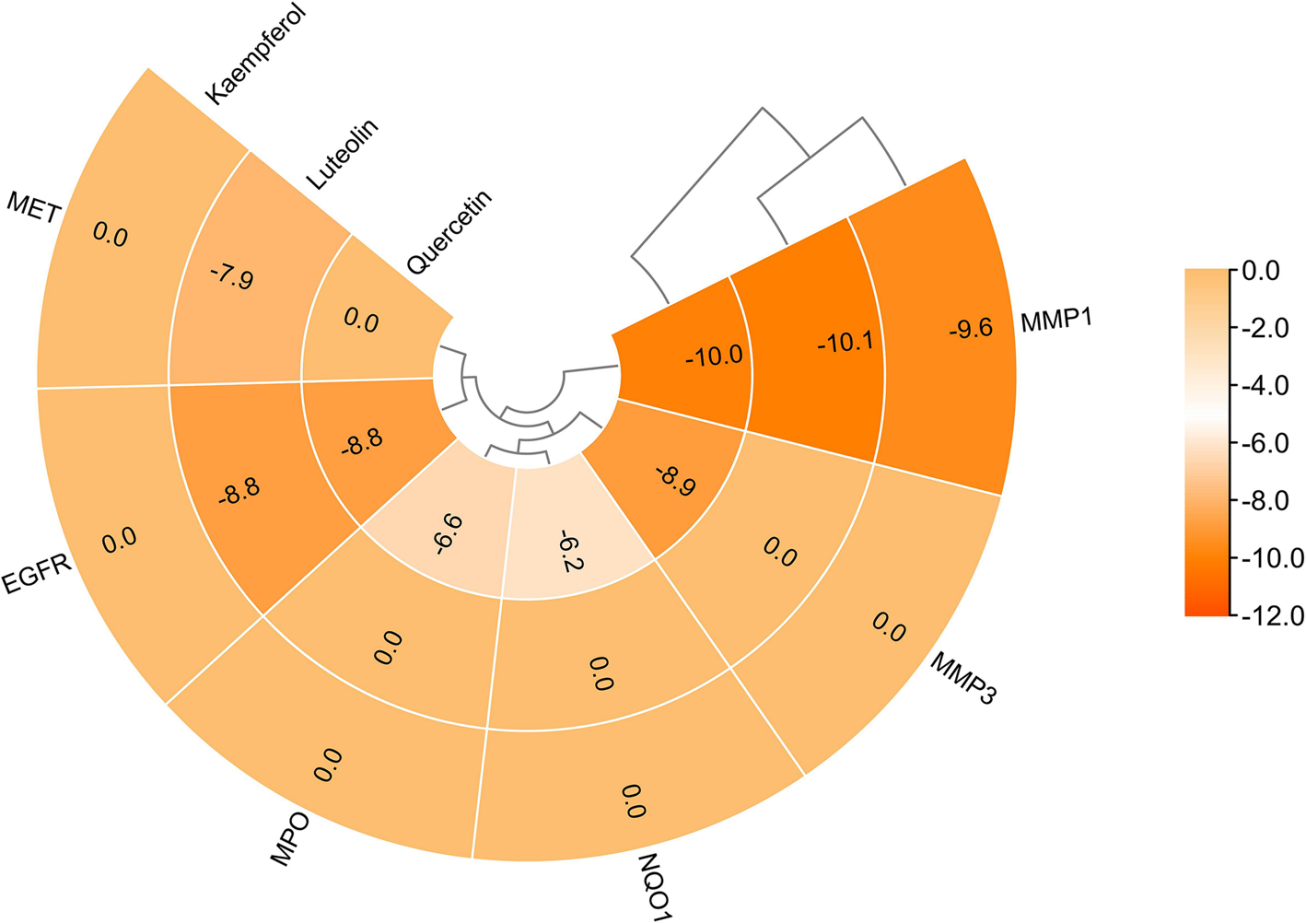
Visualization of heat map of molecular docking results, the affinity is used to represent the color range. The smaller the affinity is, the more significant the result is, color represents the value of affinity, the darker the color, the better the affinity

Based on the molecular docking results, items with affinities ranging from values (affinity ≤ -7.0 kcal/mol) were used, and the 3 SAIN components and 6 targets for which detailed results of molecular docking are given in Table 2 were selected. To visualize the results, the results are presented in detail in a Heatmap (Fig. 7).

**Table 2:** The specific molecular docking results of 3 compounds and 6 targets

Compounds	Targets	PDB ID	Resolution	Affinity (kcal/mol)	H-Bond (Dist ≤ 3.0)	UniProt ID	Method	dist from rmsd l.b	best mode rmsd u.b
Quercetin	MMP1	966C	1.90 Å	-10.0	6	P03956	X-RAY DIFFRACTION	0	0
	MMP3	1HY7	1.50 Å	-8.9	5	P08254	X-RAY DIFFRACTION	0	0
	EGFR	3W2S	1.90 Å	-8.8	3	P00533	X-RAY DIFFRACTION	0	0
	NQO1	5FUQ	2.04 Å	-6.2	2	P15559	X-RAY DIFFRACTION	0	0
	MPO	5WDJ	2.40 Å	-8.9	3	P05164	X-RAY DIFFRACTION	0	0
Luteolin	MMP1	966C	1.90 Å	-10.1	5	P03956	X-RAY DIFFRACTION	0	0
	EGFR	3W2S	1.90 Å	-8.8	2	P00533	X-RAY DIFFRACTION	0	0
	MET	4EEV	1.80 Å	-7.9	3	P08581	X-RAY DIFFRACTION	0	0
Kaempferol	MMP1	966C	1.90 Å	-9.6	5	P03956	X-RAY DIFFRACTION	0	0

### Analysis of subcellular localization prediction results

To probe the specific location in the cell of the six proteins that were screened, the PSORT II, CELLO, and BUSCA databases were used for subcellular localization predictions and predictions from three databases PSORT II (Tables 3, 4 and 5), CELLO (Tables 3, 4 and 5), and BUSCA (Tables 3, 44 and 5) were obtained.

**Table 3:** The results of PSORT II

Compounds	Protein	PDB ID	Location (k=23)	Certainty (%)	Approach	UniProt ID
Luteolin Quercetin Kaempferol	MMP1	966C	cytoplasmic mitochondrial nuclear	39.1% 26.1% 21.7%	k-NN	P03956
Quercetin	MMP3	1HY7	mitochondrial cytoplasmic	56.5% 30.4%	k-NN	P08254
Quercetin	NQO1	5FUQ	mitochondrial cytoplasmic nuclear	47.8% 21.7% 17.4%	k-NN	P15559
Quercetin Luteolin	EGFR	3W2S	cytoplasmic nuclear	69.6% 21.7%	k-NN	P00533
Luteolin	MET	4EEV	cytoplasmic nuclear mitochondrial	43.5% 30.4% 26.1%	k-NN	P08581
Quercetin	MPO	5WDJ	nuclear cytoplasmic	78.3% 13.0%	k-NN	P05164

**Table 4:** The results of CELLO

Compounds	Protein	PDB ID	Location	RELIABILITY	Approach	UniProt ID
Luteolin Quercetin Kaempferol	MMP1	966C	Extracellular Cytoplasmic	2.122* 2.074*	SVM-RFE	P03956
Quercetin	MMP3	1HY7	Cytoplasmic	3.373*	SVM-RFE	P08254
Quercetin	NQO1	5FUQ	InnerMembrane Cytoplasmic Periplasmic	1.851* 1.311* 1.209*	SVM-RFE	P15559
Quercetin Luteolin	EGFR	3W2S	Cytoplasmic Nuclear	2.510* 1.183	SVM-RFE	P00533
Luteolin	MET	4EEV	Cytoplasmic InnerMembrane	3.156* 1.166	SVM-RFE	P08581
Quercetin	MPO	5WDJ	Nuclear	1.215*	SVM-RFE	P05164

**Table 5:** The results of BUSCA

Compounds	Protein	PDB ID	Location	Score	Approach	UniProt ID
Luteolin Quercetin Kaempferol	MMP1	966C	cytoplasm	0.7	BetAware etc.	P03956
Quercetin	MMP3	1HY7	mitochondrion	1	BetAware etc.	P08254
Quercetin	NQO1	5FUQ	mitochondrion	1	BetAware etc.	P15559
Quercetin Luteolin	EGFR	3W2S	cytoplasm	1	BetAware etc.	P00533
Luteolin	MET	4EEV	cytoplasm	1	BetAware etc.	P08581
Quercetin	MPO	5WDJ	cytoplasm	0.7	BetAware etc.	P05164

By comparing the results of the three subcellular localization prediction databases, we found that the PSORT II had a higher compatibility and reliability than the other two databases. Therefore, we chose to use the prediction results of PSORT II. The database uses the k-NN algorithm (k = 23), which is currently a relatively accurate algorithm for the prediction of subcellular localization.

Subcellular localization prediction showed that MMP1, MMP3, NQO1, EGFR, MET, and MPO were found in the cytoplasm, and among them, MMP1, MMP3, NQO1, and MET were also found in the mitochondria, in addition to MMP3. The other five proteins were all found in the nucleus.

### Results of patient prognostic survival analysis of genes corresponding to the 6 targets

The purpose of survival analysis is to provide a patient with the option to continue treatment before undergoing surgery or other treatments, and is derived from the analysis of genetic data associated with the disease when the patient can survive after treatment with the corresponding target [37].

**Fig. 8:**
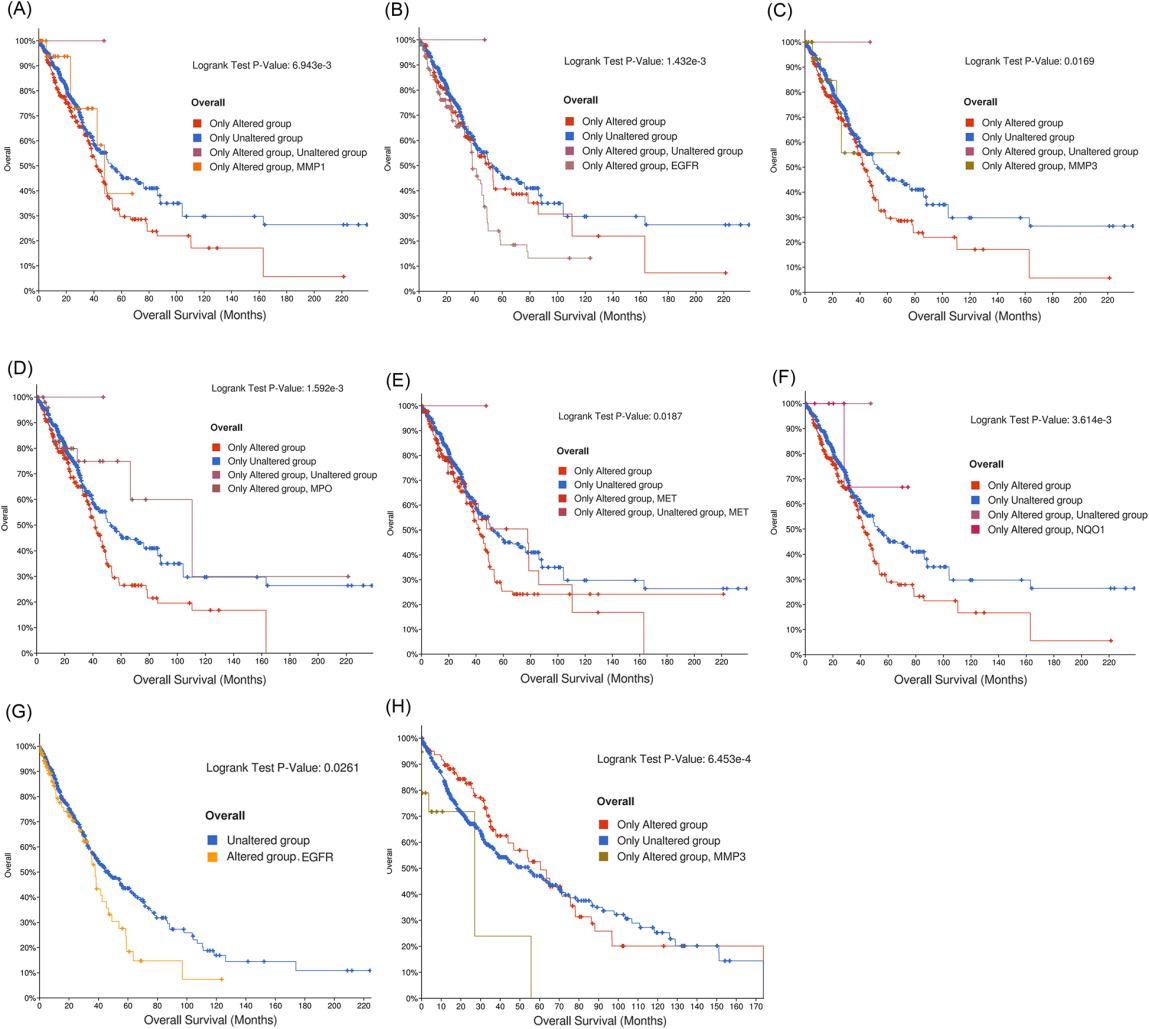
Results of prognostic survival analysis of six genes of LUAD and LUSC. A-F is the result of LUAD and g-h is the result of LUSC (**a**). Only altered MMP1 (**b**). Only altered EGFR (**c**). Only altered MMP3 (**d**). Only altered MPO (**e**). Only altered MET (**f**). Only altered NQO1 (**g**). Only altered EGFR (**h**). Only altered MMP3

Survival analysis of these six targets showed that all six genes showed statistical significance in the regulation of LUAD (P < 0.05), while only EGFR and MMP3 were statistically significant in the regulation of LUSC (P < 0.05), which was consistent with our previous TPM analysis (Fig. 8).

### Analysis of MD simulation results

**Fig. 9:**
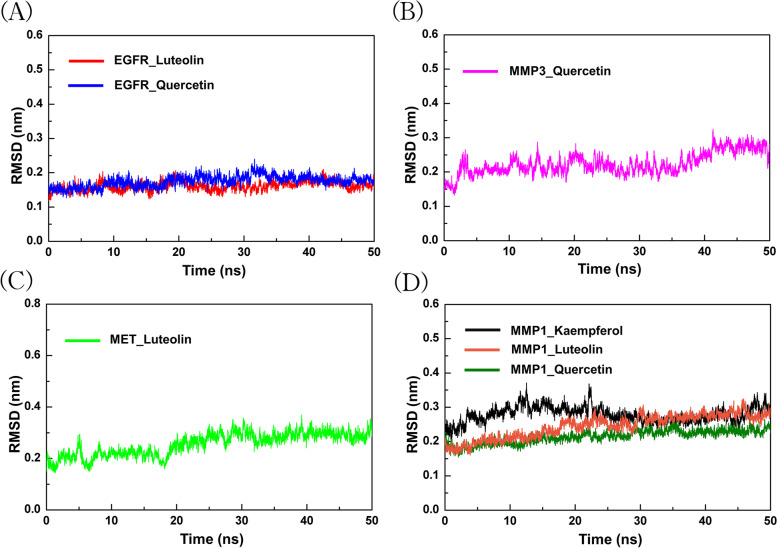
MD simulation results of seven systems (**a**). EGFR-L and Q (**b**). MMP3-Q (**c**). MET -L (**d**). MMP1-L, Q and K

RMSD data are an important basis for measuring the stability of the system and can be used to measure the stability of the tertiary structure of a protein after incorporating small molecules [38]. To make the RMSD data behave more intuitively, data were visualized (Fig. 9).

EGFR, NSCLC targets have been used in clinic, and the RMSD change trend and starting value in the time period of 0–50 ns are similar to those of the MMP3 protein. In addition, EGFR and MMP3 exhibit a very stable conformation (average RMSD < 0.15) after forming a complex under the action of L or Q. Similarly, for clinical use with the target MET, its complex formed under the action of L was relatively stable, but the MMP1 protein had a stronger structural stability than MET when in complex with L or Q. When K is in a complex with MMP1, the stability of the complex in the human setting is general, but it does not show major unusual fluctuations (Fig. 9).

Therefore, L, Q, and K, the active ingredients of SAIN, may play an important role in improving or treating NSCLC (LUAD and LUSC) after acting on the targets EGFR, MET, MMP1, and MMP3.

## Discussion

### Current status of therapeutic targets and MMP3 for LUSC

Compared with LUAD, LUSC has a unique pathological morphology (cancer cell growth location, direction, and variation) in NSCLC, therefore, no targeted therapy has been developed for LUSC due to few oncogenic aberrations that can target LUSC.

Fibroblast growth factors (FGFRs) belong to the family of receptor tyrosine kinases (RTKs) along with EGFR, which comprises the four members FGFR 1–4. In our study, we observed amplification or mutations of FGFR1-4 in NSCLC, which play a crucial role in tumor development and maintenance. In 2016, a gene sequencing study that included 4853 cancer patients revealed that 7.1% of cancer patients had abnormalities in both FGFR 1–4 genes. The most commonly found amplification of the FGFR-1 gene accounted for 50% [39]. A large body of clinical evidence has shown that FGFR-1 inhibitors play a key role in targeting the symptoms caused by LUSC, especially Infigratinib (BGJ398), BGJ398 for the treatment of LUSC, with a disease control rate of 47.6% [40]. Therefore, FGFR-1 may be the next effective target for LUSC selective therapy.

Matrix metalloproteinase 3 (MMP3) is the third member of the matrix metalloproteinase (MMP) family, and studies have shown that it plays many roles in healthy humans and patients, such as promoting epithelial mesenchymal transition, increasing the levels or activity of pulmonary profibrotic mediators, or decreasing antifibrotic mediators, and promoting abnormal epithelial cell migration and other abnormal repair processes [41]. In addition, adipocytes can increase the invasive ability of tumor cells to the body and increase the metastatic risk of lung tumors by producing exosomes with high levels of MMP3 [42, 43].

It is well known that insulin enhances the proliferation, migration, and drug resistance of NSCLC cells by activating the PI3K / Akt pathway. In previous studies, it has been shown that insulin can upregulate gene expression of MMP3 in NSCLC cells, and that its expression can be inhibited by PI3K / Akt pathway inhibitors [44].

In previous studies, it has been shown that the genes and proteins of FGFR-1 and MMP3 are highly expressed in esophageal squamous cell carcinoma, and may be associated with esophageal cancer invasion and metastasis [45].

Our findings showed that MMP3 positively regulated NSCLC, especially LUSC by participating in transcriptional misregulation in cancer, the IL-17 signaling pathway, and the rheumatoid arthritis pathway. Furthermore, molecular docking results showed that Q could bind to MMP3 protein with high binding affinity of -8.9 kcal/mol. Therefore, we speculated that there may be consistency or homology between MMP3 and FGFR-1 in treating LUSC. MMP3 may serve as a biomarker for the diagnosis and prognosis of LUSC [46]. However, more data and a large number of clinical cases are needed to verify this conclusion.

### EGFR and MET in LUAD targeted therapy

The latest NSCLC guideline 2021 V2 edition issued by the national comprehensive cancer network (NCCN) indicated that genes associated with targeted therapy of NSCLC include EGFR, KRAS, ALK, ROS1, MET, BRAF, RET, and NTRK [47]. However, these genes were mainly targeted for LUAD.

Receptor tyrosine kinases (RTKs) are high affinity cell surface receptors for many hormones, cytokines, and polypeptide growth factors. Of the 90 unique tyrosine kinase genes identified in the human genome, 58 encode RTK proteins. RTKs have not only been shown to be key regulators of normal cellular processes but also play a key role in the initiation and progression of many types of cancer [47].

EGFR and MET belong to members of the RTK family, and MET, the tyrosine kinase transmembrane receptor for MET, is a protein product encoded by the c-MET proto oncogene that has been of interest in the clinic as a potential therapeutic target in multiple tumor types [48].

Currently, a variety of targeted drugs have been developed for NSCLC targeting EGFR mutations and MET exon14 alterations, among which, drugs targeting EGFR mutations are mainly classified into two major classes: small molecule tyrosine kinase inhibitors (TKI) and monoclonal antibodies, and the TKI class mainly includes *Gefitinib*, *Erlotinib, Afatinib, Osimertinib, Dacomitinib, Lcotinib,* and *Brigatinib*, among others, monoclonal antibodies are comparatively rare and mainly include *Necitumumab, Amivantamab,* and *Ramucirumab*, of which *Amivantamab* targets the EGFR exon20 insertion [49].

Drugs targeting alterations in MET exon14 are two major classes of TKIs and monoclonal antibodies, multi kinase inhibitors, including TKIs (*Crizotinib, Cabozantinib, MGCD265, AMG208, Altiratinib, Golvatinib*) and selective MET inhibitors (*Capmatinib, Tepotinib, Tivantinib*). Monoclonal antibodies can be further divided into Anti-MET antibodies (*Onartuzumab, Emibetuzumab*) and anti-HGF antibodies (*Ficlatuzumab, Rilotumumab*).

The development of these drugs supported the results of our analysis, that is, MET gene expression in LUAD patients was much higher than that in LUSC patients, EGFR gene expression was roughly the same in the two groups of patients, and occasionally LUSC patients showed a higher expression. The results of molecular docking studies showed that SAIN's component l to Q could stably bind to the EGFR protein with an affinity of both -8.8 kcal/mol, while the MET protein could only bind to component l with an affinity of -7.9 kcal/mol. Survival analysis showed that among LUAD patients, EGFR mutated patients (P < 0.001) had significantly shorter prognostic survival relative to patients with MET mutations (P < 0.02).

### Role of MPO in NSCLC patients

Smoking confers an increased risk of lung cancer, and in an earlier study, no significant difference by NQO1 genotype was found in a large cohort of patients with combined smoking and nonsmoking lung adenocarcinoma. The data showed that smokers with combined MPO genotype had a lower lung cancer prevalence than nonsmokers [50].

MPO is a member of the heme peroxidase superfamily and is mainly expressed in neutrophils and monocytes [51]. It has been shown that MPO targeted therapy can exhibit a favorable prognostic survival status in LUAD patients [52], which is consistent with the results of our survival analysis (P < 0.001). Molecular docking studies showed that component Q of SAIN could specifically bind the MPO protein (affinity = -8.8 kcal/mol). Despite the evidence that MPO is functional in NSCLC patients, both gene differential analysis and TPM analysis results were different from that of other targets. MPO expression in tumor tissues of LUAD and LUSC was much lower compared to its expression in healthy tissues (TPM < 2), and MPO was not expressed in NSCLC patients. Based on the results from KEGG enrichment analysis, we speculated that MPO may play a role in LUAD and LUSC patients through transcriptional misregulation in cancer pathways.

High expression of MMP1 has potential in NSCLC treatment.

Molecular docking of C-T revealed that components Q, L, and K could bind to MMP1 protein with better affinity than any other component, Q (affinity = -10.0 kcal/mol), L (affinity = -10.1 kcal/mol), and K (affinity = -9.6 kcal/mol), respectively. Our survival analysis of LUAD and LUSC patients after comparing MMP1 overexpression revealed that the overexpression of MMP1 exhibited a worse prognostic survival compared with the survival time of patients after an EGFR mutation. Moreover, PPI network analysis showed that MMP1 formed three triangular structures with EGFR, MMP3, MET, and MPO (Fig. 5d), suggesting a possible link with four additional targets when MMP1 functions.

MMP1, like MMP3, belongs to the MMP family. Only few studies have been performed on MMP1 because the expression of MMP1 in rodent orthologs is not conserved [53]. However, there are several ways to study the function of MMP1 in mice and cell systems. Results from previous studies have suggested that (1) MMP1 could regulate the mechanisms of LUAD cell proliferation, migration, and invasion under the regulation of mir-202-3p [54], (2) p53 dysfunction caused by XPC gene (xeroderma pigmentosum) deficiency in lung cancer may enhance tumor metastasis by increasing MMP1 expression [55], (3) macrophage-specific inhibition of MMP1 secretion may be a potential therapy to reduce lung metastasis in smoking cancer patients [56].

Therefore, combined with the above-mentioned results, we speculate that MMP1 may be a novel target for NSCLC treatment, and l, Q, and K may be the relevant lead compounds.

### Validation of the validity of Q, L, and K

In this study, we screened the three active ingredients Q, L, and K from SAIN. However, as to whether these three ingredients have curative effects on NSCLC, experiments in cell systems or animal experiments are needed for further verification. Before performing in vitro and in vivo validation, extensive experimental validation for the treatment of NSCLC on several of these components was found in the literature, therefore, we did not perform in vitro or in vivo validation.

L (2—(3,4-dihydroxyphenyl)—5,7-dihydroxy-4-chromenone) is a natural flavonoid compound, and it has been reported that the effects of L on NSCLC are mainly reflected in (1) in lung cancer A549 cells and nuclear H460 xenograft mice. The expression of L in melanoma 2 (AIM2) was significantly reduced at the mRNA and protein expression levels, which inhibited AIM2 inflammasome activation, and in turn induced G2/M phase cell cycle arrest and inhibited the epithelial mesenchymal transition (EMT) process in NSCLC [57]. (2) At both the cellular and animal levels, L had a significant antitumor effect against NSCLC with L858R/T790M mutations in EGFR and erlotinib resistance. In addition, the mechanism is that L induces the degradation of EGFR by inhibiting the binding of Hsp90 to the mutated EGFR protein, thereby further preventing PI3K/Akt/mTOR signaling, which leads to NSCLC cell apoptosis [58, 59]. (3) At physiological concentrations, L significantly sensitizes A549 cells to the anticancer drugs oxaliplatin, bleomycin, and doxorubicin and can be used in chemotherapy as a natural sensitizer to achieve better drug availability [60, 61]. These experimental validation results are largely consistent with our findings, prediction, and MD validation results, and indicate that L has great potential for being used in the treatment of NSCLC.

Q (2—(3,4-dihydroxyphenyl)—3,5,7-trihydroxy-4-chromenone) and L are structurally similar, the only difference being that Q has more than one hydroxyl group in position 3 of the chromoenone parent nucleus. Epidemiologically, studies have shown that Q has the effect of preventing lung cancer, which is mainly reflected by (1) Q can significantly enhance tumor necrosis factor-related apoptosis inducing ligand (TRAIL)—induced cytotoxicity in NSCLC cells, thereby accelerating the death of NSCLC cells [62, 63]. (2) Priming H520 cells (LUSC cells) with Q increased cisplatin-induced apoptosis by 30.2%, and this process was accompanied by Q-induced expression of multiple apoptosis-related genes, which could serve as a potent chemosensitizer [64]. (3) Q can increase or decrease the expression of many miRNAs or TKIs, including increasing the expression of mir-16-5p, a member of the mir-16 family, decreasing the expression of WEE1, and inhibiting the expression of the SRC family [65]. Similarly, our prediction was that Q might treat or alleviate disease symptoms by decreasing the expression of MMP3, EGFR, and MMP1 in NSCLC tissues.

In previous studies, it was shown that dietary flavone K (3,5,7-trihydroxy-2—(4-hydroxyphenyl)—4-chromenone) can effectively prevent and treat lung cancer, mainly through the following mechanisms: (1) K inhibits the apoptosis of H460 cells (NSCLC cells) by inducing the overexpression of tumor suppressor gene antioxidant enzyme (Sod-2) [66]. (2) K mediates Smad3 phosphorylation at threonine 179 (THR-179) by inhibiting AKT1, which in turn inhibits transforming growth factor- β 1(TG-β 1)- induced epithelial to mesenchymal transition and migration of A549 lung cancer cells [67]. (3) K sensitizes otherwise chemoresistant cancer cells to multitarget antifolate (MTA) by inhibiting the EMT signaling pathway, which enables K to reverse the ability of NSCLC to resist MTA, thereby realizing the role of inhibiting lung cancer chemotherapy responses through the EMT pathway [68]. Our results that show that K exerts its anti NSCLC effects by inhibiting the MMP1 mediated IL-17 signaling pathway, rheumatoid arthritis pathway, and relaxin signaling pathway are consistent with our docking results and MD results.

## Conclusion

It is well known that the complexity of NSCLC etiology does not allow us to explain it by the alteration of a single gene, therefore. The treatment of lung cancer is not achieved by one means, but requires combination therapy of multiple means and multiple targets. Although the three components Q, L, and K in SAIN can do so by binding to MMP1, MMP3, EGFR, and MET, and are capable of acting as inhibitors by participating in EGFR tyrosine kinase inhibitor resistance, adherens junction, IL-17 signaling pathway, NSCLC, and the relaxin signaling pathway, exerting their functions together to prevent and treat NSCLC (LUAD and LUSC). Nobody can predict which genetic mutations are responsible for NSCLC, and therefore, a concerted effort from researchers in all fields is required for the complete cure of NSCLC.

## Data Availability

The datasets generated and/or analysed during the current study are not publicly available due the limited scope of data availability, these data are used under the license of this study and are not disclosed,but are available from the author[sedate@stu. shzu.edu.cn (Dongdong Zhang)] on reasonable request.

## Abbreviations

SAIN: Saussurea involucrate
NSCLC: Non-small cell lung cancer
LUAD: Lung adenocarcinoma
LUSC: Lung squamous cell carcinoma
SI: Snow lotus
Q: Quercetin
L: Luteolin
K: Kaempferol
H: Hispidulin
A: Alloisoimperatorin
F: Flazin
TCMSP: Traditional Chinese Medicine Systems Pharmacology Database
OB: Oral bioavailability
DL: Drug-like
GDC: Genomic Data Commons
GEO: Gene Expression Omnibus
GEPIA: Gene Expression Profiling Interactive Analysis
TCGA: The Cancer Genome Atlas
FDR: False discovery rate
GO: Gene Ontology
BP: Biology Progress
KEGG: Kyoto Encyclopedia of Genes and Genomes
PPI: Protein–Protein Interaction
Dist: Distance
IL-17: Interleukin-17
MMP1: Matrix metalloproteinase 1
MMP3: Matrix metalloproteinase 3
MET: Mesenchymal to epithelial transition factor
EGFR: Epidermal growth factor receptor
MPO: Myeloperoxidase
NQO1: Nad(p)h: quinone oxidoreductase 1
PTGS2: Prostaglandin G/H synthase 2
HSP90AA1: Heat Shock Protein 90 Alpha Family Class A Member 1
TPM: Transcripts Per Million
ALK: Anaplastic Lymphoma Kinase
ROS1: C-ros oncogene 1, receptor tyrosine kinase
C-T-P: Compounds-Targets-Pathways
cNLS: Classic nuclear localization signal
k-NN: K-Nearest Neighbor
SVM-RFE: Support vector machine recursive feature elimination algorithm
STP: Swisstargetprediction
ANOVA: Analysis of Variance
PDB: Palm DataBase
FGFR: Fibroblast growth factor
RTK: Receptor tyrosine kinase
KRAS: V-Ki-ras2 Kirsten ratsarcoma viral oncogene homolog
BRAF: V-raf murine sarcoma viral oncogene homolog B1
NTRK: Neurotrophic factor receptor tyrosine kinase
HGF: Hepatocyte growth factor
TKI: Tyrosine kinase inhibitors
AIM2: Melanoma 2
EMT: Epithelial-mesenchymal transition
XPC: Xeroderma pigmentosum
MTA: Multi-target antifolate
TMBB: Transmembrane beta-barrels
